# Effects of shortening velocity on the stiffness to force ratio during isometric force redevelopment suggest mechanisms of residual force depression

**DOI:** 10.1038/s41598-023-28236-5

**Published:** 2023-01-18

**Authors:** Siwoo Jeong, Kiisa Nishikawa

**Affiliations:** 1 Department of Physical Therapy, College of Medical Science, Jeonju University, Jeonju, South Korea; 2 Department of Biological Sciences, Northern Arizona University, Flagstaff, AZ USA

**Keywords:** Physiology, Bone quality and biomechanics

## Abstract

Although the phenomenon of residual force depression has been known for decades, the mechanisms remain elusive. In the present study, we investigated mechanisms of residual force depression by measuring the stiffness to force ratio during force redevelopment after shortening at different velocities. The results showed that the slope of the relationship between muscle stiffness and force decreased with decreasing shortening velocity, and the y-intercept increased with decreasing shortening velocity. The differing slopes and y-intercepts indicate that the stiffness to force ratio during isometric force redevelopment depends on the active shortening velocity at a given muscle length and activation level. The greater stiffness to force ratio after active shortening can potentially be explained by weakly-bound cross bridges in the new overlap zone. However, weakly-bound cross bridges are insufficient to explain the reduced slope at the slowest shortening velocity because the reduced velocity should increase the proportion of weakly- to strongly-bound cross bridges, thereby increasing the slope. In addition, if actin distortion caused by active shortening recovers during the force redevelopment period, then the resulting slope should be similar to the non-linear slope of force redevelopment over time. Alternatively, we suggest that a tunable elastic element, such as titin, could potentially explain the results.

## Introduction

Residual force depression refers to the reduction in steady-state isometric force following active shortening and force redevelopment, compared to the purely isometric force at the same final length^1–5^. The phenomenon has been systematically observed in human movement^6^, whole muscle preparations^1,5,7,8^, single fibers^9–12^, and single myofibrils^13^. Residual force depression is inversely related to shortening velocity^1,5,7^ and directly related to concentric muscle work^5,14–16^.

Several mechanisms have been proposed to explain residual force depression^17,18^. Some proposed mechanisms, including sarcomere length non-uniformity and accumulation of waste products such as inorganic phosphate, appear unlikely because they fail to predict one or more important features of residual force depression^17^. Two classes of theories have emerged as having greater explanatory power. These include inhibition of cross-bridge binding by various mechanisms related to deformation of thin filaments during active shortening^5,17^, and mechanisms related to activation-dependent stiffness of elastic elements such as titin^19–23^. However, the fundamental mechanisms for residual force depression remain unclear, and multiple mechanisms appear likely to contribute^17^.

Maréchal and Plaghki^5^ suggested that cross bridges may fail to form, or those that form may fail to produce force, in the new overlap zone between actin and myosin that forms during active shortening. These inhibited cross bridges, similar to weakly-bound cross bridges^24^, may contribute to stiffness without producing force, and therefore might also contribute to residual force depression. In contrast, strongly-bound cross bridges should contribute equally to muscle force and stiffness^10^, while super-relaxed cross bridges contribute to neither quantity^25^. Many studies have reported that muscle stiffness (*k*_*m*_) is reduced in the force-depressed state following active shortening and force redevelopment^4,8,10,26–30^. Most of these studies also found that muscle force and stiffness decreased by similar proportions in the steady state following active shortening and isometric force (*F*_*ISO*_) redevelopment, consistent with a decrease in the number of strongly attached cross bridges, assuming a proportional contribution of these cross bridges to *k*_*m*_ and *F*_*ISO*_ during isometric contraction at a given length and activation level^31–33^.

However, Julian and Sollins^34^ observed that shortening at different velocities changes the *k*_*m*_ to *F*_*ISO*_ ratio because *F*_*ISO*_ decreases more rapidly with shortening velocity than *k*_*m*_^34^. Furthermore, Joumaa et al.^4^ found that the ratio of *k*_*m*_ to *F*_*ISO*_ is greater during isometric force redevelopment than in a purely isometric contraction at the same length because active shortening decreases *F*_*ISO*_ more than *k*_*m*_. Since the ratio of *k*_*m*_ to *F*_*ISO*_ decreases proportionally following isometric force redevelopment, and since active shortening decreases *F*_*ISO*_ more than *k*_*m*_, the ratio of *k*_*m*_ to *F*_*ISO*_ should decrease during isometric force development after active shortening. Mechanisms for residual force depression could potentially be inferred by observing how the stiffness (*k*_*m*_) to force (*F*_*ISO*_) ratio changes during the isometric force redevelopment period following active shortening. To our knowledge, this question has not been investigated previously.

The larger ratio of *k*_*m*_ to *F*_*ISO*_ during force redevelopment^4^ cannot be explained solely by a reduced number of strongly-bound cross bridges in the newly formed overlap zone since the contribution of strongly bound cross bridges to both *F*_*ISO*_ and *k*_*m*_ depends linearly on the number of strongly-bound cross bridges. While many structures contribute to muscle stiffness in addition to cross bridges^37^, including thick^35^ and thin filaments^36^, only cross bridges and titin^21^ appear to exhibit activation-dependent stiffness. The stiffness of thick^35^ and thin filaments^38^ is independent of muscle activation, making any contribution to residual force depression unlikely.

During the force redevelopment period, several different relationships between *k*_*m*_ and *F*_*ISO*_ are possible. Either weakly-bound cross bridges or tunable elastic elements could increase the ratio of *k*_*m*_ to *F*_*ISO*_ during isometric force redevelopment after active shortening. Weakly-bound cross bridges that contribute to *k*_*m*_ without producing force could increase *k*_*m*_ for the same *F*_*ISO*_. The greater ratio of *k*_*m*_ to *F*_*ISO*_ could also be associated with changes in stiffness of tunable elastic elements^19^, namely titin^21–23,39^. Rassier and Herzog^18^ suggested that the equilibrium length (zero force) of a parallel element is adjusted when force redevelops isometrically following active shortening. They showed not only that the amount of force enhancement is affected by active shortening prior to active lengthening, but also that the effect of shortening on force enhancement is reduced by increasing the duration of a pause between shortening and lengthening. The parallel element is likely to be titin^40^. If titin is assumed to be a linear spring whose force is related to the amount of force enhancement^21,40,41^, then increasing force enhancement with pause duration could be associated with increased titin force and stiffness caused by adjustment of titin equilibrium length.

**Figure 1:**
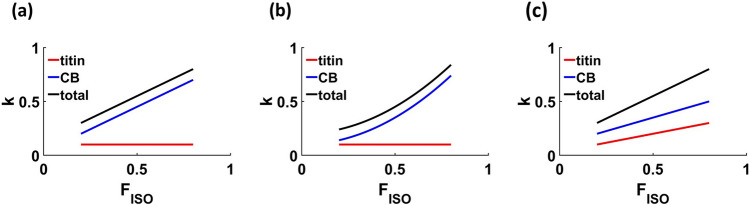
Theoretical relationships between relative muscle stiffness (*k*_*m*_) and relative isometric force (*F*_*ISO*_). Total muscle stiffness (black) is estimated as the sum of titin stiffness (red) and cross-bridge (CB) stiffness (blue). (**a**) If the proportion of weakly- to strongly-bound cross bridges is independent of *F*_*ISO*_ and titin stiffness is constant, then the relationship between *k*_*m*_ and *F*_*ISO*_ will be linear. (**b**) If titin stiffness is constant, but the proportion of weakly- to strongly-bound cross bridges depends non-linearly on *F*_*ISO*_, then the total stiffness will also increase non-linearly with *F*_*ISO*_. (**c**) If both titin stiffness and the proportion of weakly- to strongly-bound cross bridges increase linearly with *F*_*ISO*_, then the total stiffness should also increase linearly with *F*_*ISO*_.

The slope and intercept of the relationship between *k*_*m*_ and *F*_*ISO*_ during isometric force redevelopment may provide insights about how cross bridge and/or titin stiffness change during force redevelopment after active shortening. The y-intercept represents *k*_*m*_ at zero *F*_*ISO*_ and the slope of the relationship indicates how *k*_*m*_ changes with increasing *F*_*ISO*_. A non-zero y-intercept could be related to either the proportion of weakly-bound cross bridges (which contribute only to *k*_*m*_ and may represent the amount of deformation of thin filaments^5^), to titin stiffness, or both. The slope of the relationship could represent how the proportion of weakly- to strongly-bound cross bridges and/or titin stiffness change as isometric force redevelops following active shortening (Fig. 1). If the proportion of weakly- to strongly-bound cross bridges is independent of *F*_*ISO*_ and titin stiffness is constant, or alternatively if titin stiffness depends linearly on *F*_*ISO*_, then the slope should be linear (Fig. 1a). A non-linear slope could be due to either a changing proportion of strongly-bound cross bridges and/or non-linear dependence of titin stiffness on *F*_*ISO*_. If titin stiffness is constant, but the proportion of weakly- to strongly-bound cross bridges depends non-linearly on *F*_*ISO*_, then the total stiffness should increase non-linearly in direct proportion with *F*_*ISO*_ (Fig. 1b). If both titin stiffness and the proportion of weakly- to strongly-bound cross bridges increase linearly with *F*_*ISO*_, then the total stiffness should increase linearly with *F*_*ISO*_ (Fig. 1c). A non-linear relationship between *k*_*m*_ and *F*_*ISO*_ that mirrors force redevelopment with zero intercept (Fig. 1b) would be consistent with a cross-bridge-based mechanism for residual force depression. However, a linear relationship between *k*_*m*_ and *F*_*ISO*_ with a non-zero intercept (Fig. 1c) would suggest alternative mechanisms, including a mechanism in which titin stiffness depends linearly on force.

The aim of this study was to systematically investigate how the ratio of *k*_*m*_ to *F*_*ISO*_ changes during isometric force redevelopment following active shortening at different velocities by measuring the *k*_*m*_ to *F*_*ISO*_ ratio at varying times after the onset of isometric force redevelopment. In contrast, previous studies measured the *k*_*m*_ to *F*_*ISO*_ ratio at a single time point^4,8^. We designed an experiment in which muscles first develop force isometrically, and then shorten at different velocities, followed by varying durations of isometric force redevelopment during which *F*_*ISO*_ and *k*_*m*_ were measured.

## Results

### Residual force depression

**Figure 2:**
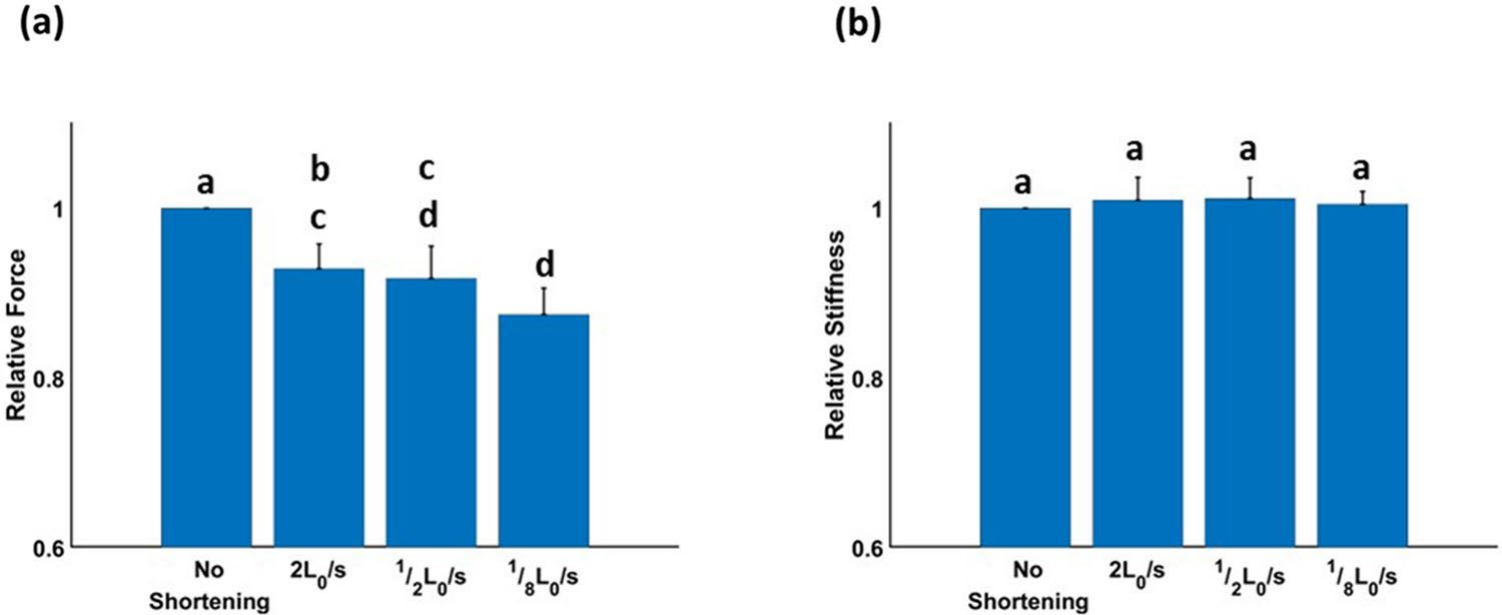
Relative isometric force and stiffness after 1000 ms of active shortening. (**a**) Mean relative isometric forces after active shortening were lower than pure isometric force (HSD, *p* < 0.05). The fastest active shortening velocity produced a larger mean relative force than the slowest active shortening velocity (HSD, *p* < 0.05), demonstrating less residual force depression in the fastest active shortening velocity than in the slowest active shortening velocity. (**b**) Mean relative muscle stiffness after active shortening did not differ significantly among shortening velocities (ANOVA, F = 0.37, *p* < 0.77). Bars with the same letter are not significantly different (HSD, *p* > 0.05).

After 1000 ms of isometric force redevelopment, relative muscle force (*F*_*ISO*_) differed significantly among shortening velocities (Fig. 2a, ANOVA, F = 19.91, *p* < 0.001), and was significantly lower than *F*_*ISO*_ with no active shortening (Fig. 2a, HSD, *p* < 0.05), indicating that residual force depression had occurred. The slowest active shortening velocity (1/8 L_0_/s) generated a significantly lower isometric force (0.87 ± 0.01 *F*_*ISO*_ at 1000 ms) than the fastest active shortening velocity (2L_0_/s, 0.93 ± 0.01 *F*_*ISO*_ at 1000 ms), but the intermediate velocity (1/2L_0_/s, 0.92 ± 0.02 *F*_*ISO*_ at 1000 ms) did not differ significantly from the other shortening velocities (1/8 L_0_/s and 2 L_0_/s). In contrast to *F*_*ISO*_, *k*_*m*_ did not differ significantly among shortening velocities after 1000 ms of isometric force redevelopment (Fig. 2b, ANOVA, F = 0.37, *p* = 0.77).

### F_ISO_ and k_m_ following active shortening

**Figure 3:**
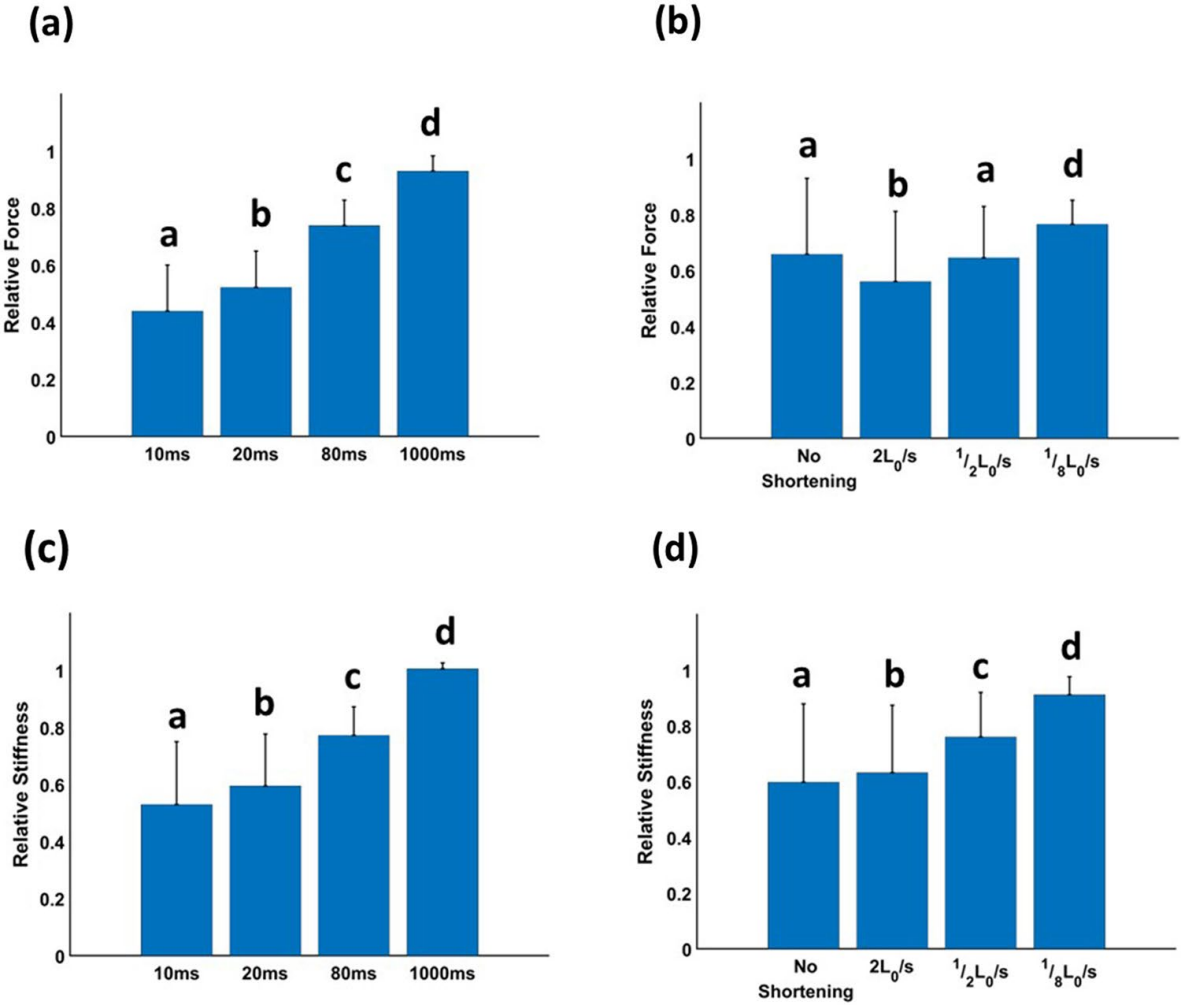
Mean values (+ 1 S.D.) of relative force (**a, b**) and relative stiffness (**c, d**) after different pause durations (**a, c**) and different velocities of active shortening (**b, d**). Mean relative force (**a**) and stiffness (**c**) represent mean values for all conditions including no shortening, 1/8L_0_/s, 1/2L_0_/s and 2L_0_/s, and mean relative force (**b**) and stiffness (**d**) represent mean values for all pause durations including 10, 20, 80, and 1000 ms. Mean relative force and stiffness increased with increasing pause duration and decreasing shortening velocity. Bars with the same letter are not significantly different (HSD, *p* > 0.05).

**Figure 4:**
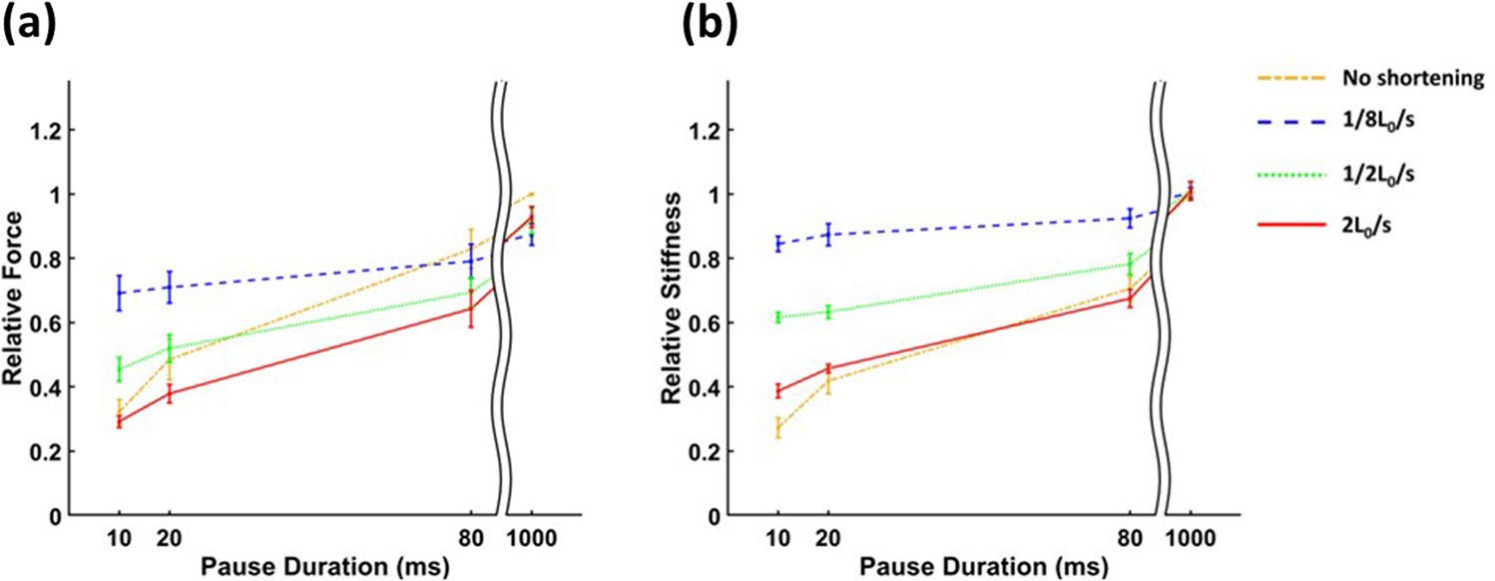
Change in relative muscle force and stiffness during isometric force redevelopment following active shortening at different velocities. Data points (n = 7 muscles per group) are means across muscles with error bars representing S.D. (**a**) Relative force increases with increasing pause duration. The rate of increase varies with shortening velocity. (**b**) Relative stiffness increases with increasing pause duration and shortening velocity affects the stiffness vs. pause duration relationship. The slope of the relationship increases with increasing shortening velocity.

**Figure 5:**
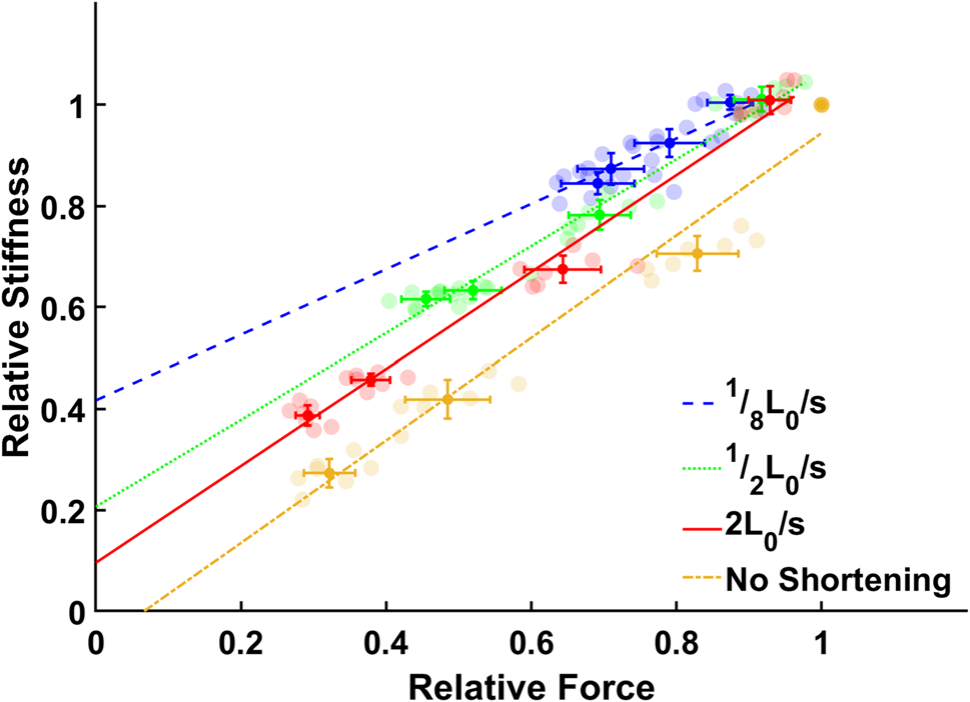
Relative force vs. relative stiffness at different active shortening velocities. Data points (n = 7 muscles per group) are means across muscles with error bars representing S.E. The slope and the y-intercept of the relative stiffness-force relationship differed significantly among shortening velocities, including ‘no shortening’ (ANCOVA, slope, *p* < 0.001; y-intercept, *p* < 0.001).

Both *F*_*ISO*_ and muscle stiffness (*k*_*m*_) increased during the period of isometric force redevelopment following active shortening (Fig. 3a, c). The rate of increase in both *F*_*ISO*_ and *k*_*m*_ during the force redevelopment period depended on shortening velocity (Fig. 4). The ratio of *k*_*m*_ to *F*_*ISO*_ also increased with decreasing shortening velocity, showing different y-intercepts and slopes for different shortening velocities (Fig. 5).

ANOVA demonstrated significant effects of shortening velocity (*p* < 0.001), pause duration (*p* < 0.001), and their interaction (*p* < 0.001) on *F*_*ISO*_ (Table 1). All pause duration group means and shortening velocity group means were significantly different from each other except for ‘no shortening’ and ‘1/2 L_0_/s’ (Fig. 3a, b, HSD, *p* < 0.05). *F*_*ISO*_ increased with increasing pause duration and decreasing shortening velocity. For the *F*_*ISO*_-pause duration relationship, the slope of ‘no shortening’ was the steepest and the slopes of the active shortening group varied with shortening velocity (Fig. 4a).

**Table 1:** Results of two-way ANOVA. Effects of pause duration (10, 20, 80 and 1000 ms) and shortening velocity (1/8, 1/2, and 2 L0/s) on relative muscle force.

	Df	Sum Sq	Mean Sq	F-value	*p* value
Pause duration	3	4.09	1.36	712.78	< 0.001
Shortening velocity	3	0.60	0.20	104.26	< 0.001
Interaction	9	0.71	0.08	41.03	< 0.001
Error	96	0.18	0.0019		
Total	111	5.58			

The effects of shortening velocity (*p* < 0.001), pause duration (*p* < 0.001), and interaction (*p* < 0.001) on *k*_*m*_ were also significant (Table 2). All pause duration and shortening velocity group means were significantly different from each other (Fig. 3c, d, HSD, *p* < 0.05). The rate of increase in *k*_*m*_ during the force redevelopment period depended on shortening velocity. As for *F*_*ISO*_, the slope of ‘no shortening’ was the steepest and the slopes of the active shortening group increased with increasing shortening velocity (Fig. 4b).

**Table 2:** Results of two-way ANOVA. Effects of pause duration (10, 20, 80 and 1000 ms) and shortening velocity (1/8, ½, and 2 L_0_/s) on relative stiffness.

	Df	Sum Sq	Mean Sq	F-value	*p*-value
Pause duration	3	3.81	1.27	1767	< 0.001
Shortening velocity	3	1.70	0.57	787.79	< 0.001
Interaction	9	0.82	0.09	126.66	< 0.001
Error	96	0.07	0.00072		
Total	111	6.39			

### The relationship between *F*_*ISO*_ and *k*_*m*_

The slopes and y-intercepts of the relationship between *F*_*ISO*_ and *k*_*m*_ differed significantly among shortening velocities (Fig. 5, ANCOVA, F = 134.29, *p* < 0.001, y-intercept; ANCOVA, F = 6.71, *p* < 0.001, slope). At the slowest shortening velocity (1/8 L_0_/s; Fig. 5, blue), *k*_*m*_ increased more slowly with *F*_*ISO*_ than for the faster velocities (1/2 L_0_/s, Fig. 5, green, *p* < 0.05; 2 L_0_/s, Fig. 5, red, *p* < 0.001) and also more slowly than for the ‘No shortening’ condition (Fig. 5, yellow, *p* < 0.001). All y-intercepts were significantly different from each other (Table 3). A significant positive correlation between *F*_*ISO*_ and *k*_*m*_ was observed for all active shortening velocities as well as for ‘No shortening’ (Table 3, *p* < 0.001).

**Table 3:** Slope, intercept (with 95% confidence bounds) and correlation coefficients for the linear relationship between muscle force and stiffness after shortening at three velocities (1/8, 1/2 and 2 L_0_/s) and with no shortening.

Shortening velocity	Slope	Intercept	Correlation
No shortening	1.01 (0.93, 1.09)	− 0.067 (− 0.12, − 0.012)	0.98*
1/8 L_0_/s	0.65^a,b^ (0.48, 0.82)	0.42^c^ (0.29, 0.55)	0.84*
1/2 L_0_/s	0.86 (0.79, 0.93)	0.21^c,d^ (0.17, 0.26)	0.98*
2 L_0_/s	0.96 (0.90, 1.02)	0.10^b,c,e^ (0.062, 0.14)	0.99*

^a^Significantly smaller than ‘No shortening’ (*p* < 0.01).

^b^Significantly smaller than ‘1/2 L_0_/s’ (*p* < 0.01).

^c^Significantly larger than ‘No shortening’ (*p* < 0.001).

^d^Significantly smaller than ‘1/8 L_0_/s’ (*p* < 0.05).

^e^Significantly smaller than ‘1/8 L_0_/s’ (*p* < 0.001).

*p < 0.001.

## Discussion

The aim of this study was to investigate mechanisms of residual force depression by observing how the ratio of muscle stiffness (*k*_*m*_) to force (*F*_*ISO*_) changes during the period of isometric force redevelopment following active shortening at different velocities. Our main findings were that: (1) *F*_*ISO*_ and *k*_*m*_ increased during the force redevelopment period following active shortening (Tables 1 and 2, Fig. 3); (2) the rate of increase in *F*_*ISO*_ and *k*_*m*_ was affected by the active shortening velocity (Fig. 4); and (3) the slope and y-intercept of the relationship between *F*_*ISO*_ and *k*_*m*_ differed among shortening velocities (Table 3 and Fig. 5).

Residual force depression occurred during the isometric force redevelopment period following active shortening (Fig. 2a). All values of *F*_*ISO*_ at 1000 ms following active shortening were lower than *F*_*ISO*_ at 1000 ms without active shortening, and *F*_*ISO*_ following the fastest active shortening velocity was higher than *F*_*ISO*_ following the slowest velocity (Fig. 2a). The results are consistent with previous studies which found that the amount of residual force depression is inversely proportional to shortening velocity ^1,5,7^. We also found that, following active shortening, all values of *k*_*m*_ during isometric force redevelopment recovered to a value corresponding to *k*_*m*_ with no active shortening after a pause duration of 1000 ms (Fig. 2b). This result is not consistent with a previous study^27^, which found that the amount of force depression was positively correlated with the amount of stiffness depression in whole muscle. Although Corr and Herzog^8^ found that the amount of force depression decreased with increasing shortening velocity, stiffness depression was independent of shortening velocity over a range of velocities between ~ 7 and 62% L_0_/s. In addition, in muscle fibers^42^, force depression was weakly related to stiffness depression when force depression was small (< 15%, see Fig. 4 in Ref^42^). Therefore, the relatively small magnitude of force depression observed in the present study likely explains the different results.

The y-intercept of the relationship between *F*_*ISO*_ and *k*_*m*_ increased significantly with decreasing active shortening velocity, and the y-intercept for ‘no shortening’ was significantly lower than for the experiments with active shortening (Table 3 and Fig. 5). The velocity-dependent y-intercept suggests that *k*_*m*_ is substantial when *F*_*ISO*_ is zero, and that the stiffness to force ratio is not constant for isometric contractions following active shortening. This result is similar to results of previous studies that measured *k*_*m*_ during the isometric force redevelopment period following active shortening ^26,34^. Julian and Sollins^34^ varied shortening velocity to investigate the stiffness to force ratio (see Fig. 6 in Ref^34^), and also found a substantial y-intercept for the relationship. Julian and Morgan^26^ found that force decreased faster than *k*_*m*_ during a tension transient (see their Fig. 2 in Ref^26^). Corr and Herzog^8^ found that *k*_*m*_ decreased during shortening compared to the isometric value, but they found no difference in *k*_*m*_ among shortening velocities over a smaller range of shortening velocities that included 7%, 21% and 63% L_0_ per second^8^. A cross-bridge model predicted increasing *k*_*m*_ with increasing shortening velocity over the same range of velocities^27^.

**Figure 6:**
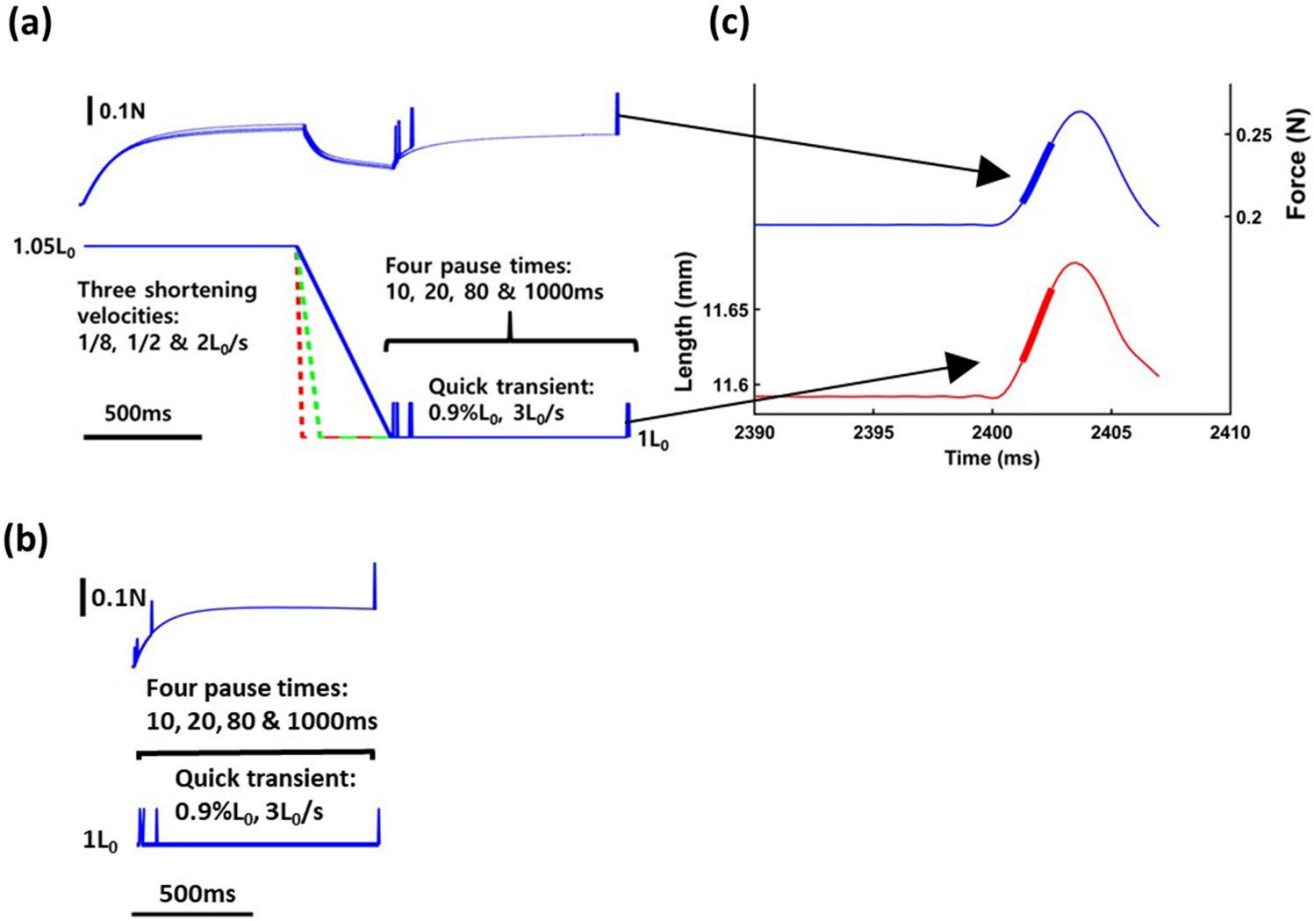
Muscle force (**a**, upper trace) and length (**a**, lower trace) in the active shortening group, muscle force (**b**, upper trace) and length (**b**, lower trace) in the pure isometric group and measurement of instantaneous muscle stiffness (**c**). (**a**) Protocols used to evaluate the effects of active shortening and duration of isometric force redevelopment on muscle stiffness and force. Active shortening was performed from 1.05L_0_ to L_0_ at three different velocities; 1/8 L_0_/s (red), ½ L_0_/s (green) and 2 L_0_/s (blue). Instantaneous muscle stiffness was measured using a quick transient with an amplitude of 0.9% L_0_ and a velocity of 3L_0_/s for four pause durations (10, 20, 80 and 1000 ms). (**b**) Pure isometric contraction was performed at L_0_. Instantaneous muscle stiffness was measured using a quick transient for four pause duration (10, 20, 80 and 1000 ms). (**c**) Muscle force (upper trace, blue) responded to a rapid transient (lower trace, red) at 3L_0_/s. The linear parts of the force–time trace (thick line, blue) and the length–time trace (thick line, red) were used to determine stiffness. The linear slope of the force–time trace was divided by the linear slope of the length–time trace, which gives the stiffness of muscle (N/mm) at steady state.

According to the cross-bridge theory, *k*_*m*_ should be zero when force is zero because *F*_*ISO*_ depends on the number of attached cross bridges^31,32^. As expected, *k*_*m*_ is close to 0 when *F*_*ISO*_ equals zero for the ‘no shortening’ protocol (Fig. 5, yellow). However, the ‘active shortening’ protocols showed non-zero y-intercepts that differed among the three shortening velocities (Fig. 5, blue, green and red). These results are not consistent with the cross-bridge theory^33,43^. In order to explain the non-zero y-intercepts caused by active shortening, Maréchal and Plaghki^5^ suggested that cross bridges in the newly forming overlap zone should produce no force but contribute to stiffness, similar to weakly bound cross bridges^24^. As the weakly-bound cross bridges contribute only to *k*_*m*_ without producing force^4,37^, the non-zero y-intercepts in the present study could potentially be explained by weakly-bound cross bridges. Previous studies found that residual force depression decreases with increasing shortening velocity^5,7,17,44^, and proposed that the reduction of force is associated with the amount of stress imposed on actin filaments during active shortening ^4^ or with the amount of mechanical work produced by the muscle during active shortening^3,45,46^. According to this hypothesis, the reduced stress on actin filaments caused by faster shortening (i.e., lowest work) results in binding of a smaller number of weakly-bound cross bridges to actin, which results in diminished residual force depression. The number of weakly-bound cross bridges increases with increasing actin stress produced by decreasing shortening velocity. Similar to these previous studies, the present study also found that that the slowest velocity (i.e., greatest work) resulted in the largest y-intercept between *k*_*m*_ and *F*_*ISO*_, and also found that residual force depression decreases with increasing shortening velocity. These observations are potentially consistent with the hypothesis that more weakly-bound cross bridges are bound to actin when the shortening velocity is relatively slow.

In terms of the relationship between *k*_*m*_ and *F*_*ISO*_, the three shortening velocities had different slopes during isometric force redevelopment (Fig. 5). If the velocity-dependent y-intercept indicates the number of weakly-bound cross bridges, then the different slopes suggest a different proportion of weakly- to strongly-bound cross bridges in the newly formed overlap zone. If the lower active shortening velocity results in greater stress and distortion of actin^4,5^, this would increase the proportion of weakly-bound cross bridges. The greater distortion of actin developed by the slower active shortening velocity should increase the proportion of weakly- to strongly-bound cross bridges. The greater proportion should increase the slope because weakly-bound cross bridges contribute only to *k*_*m*_ without producing force. Therefore, while the ‘weakly-bound cross bridge’ mechanism can potentially explain the velocity-dependent y-intercept, it is not consistent with the reduced slope at the slower active shortening velocity. In contrast to a mechanism involving weakly-bound cross bridges, inhibition of strongly-bound cross bridges by tropomyosin distortion or other mechanisms^17^ should not affect the stiffness to force ratio^4^.

Another problem with the weakly-bound cross-bridge hypothesis is that the stiffness must increase faster than linearly during force redevelopment after active shortening, as weakly-bound cross bridges are converted to the strongly bound state. In principle, any function (line, higher order polynomial, and exponential) can be fit to a line with high *r*^2^, given appropriate values for the coefficients. However, a line will not provide a good fit to sufficiently non-linear data. The fact that the linear function fit our data as well as the other functions therefore supports a linear relationship. To produce the linear relationship between *k*_*m*_ and *F*_*ISO*_ observed during force redevelopment (Fig. 5), the proportion of weakly- to strongly-bound cross bridges must be constant, and therefore the amount of actin distortion must also be constant during force redevelopment (see Fig. 1). Actin distortion has not been directly quantified after active shortening. Yet, previous studies have suggested that the amount of actin distortion depends on the amount of stress or work performed during shortening^5,30^. During the period of force redevelopment after shortening, the amount of actin distortion developed by active shortening should not be constant since the stress is not constant and work is zero. The distorted actin filament should return toward its original state to some extent during the period of force redevelopment, and the proportion of weakly- to strongly-bound cross bridges should decrease. However, we observed that the slope was constant throughout the force redevelopment period except at the slowest velocity (Table 3), which suggests that actin distortion does not affect the proportion of weakly- to strongly- bound cross bridges. Therefore, the linear *F*_*ISO*_ – *k*_*m*_ relationship cannot be explained solely by weakly-bound cross bridges and an alternative hypothesis is needed.

A role for an elastic element such as titin in residual force depression and residual force enhancement has been suggested in previous studies^19–23^. Rassier and Herzog^18^ showed that active shortening prior to active lengthening decreases the amount of residual force enhancement, and also that increasing the pause duration between shortening and lengthening reduces the effect of active shortening on the amount of residual force enhancement. If titin contributes to residual force enhancement^21,41,47–49^, then titin force following active lengthening should be affected by active shortening and the pause duration between shortening and lengthening. A recent study also demonstrates that muscle passive tension increases100-fold upon activation^50^. This large increase in passive tension of the I-band could readily account for the increased force associated with force enhancement as well as the decrease in force due to residual force depression, assuming that active shortening reduces the tension of this passive component. Simulation-based studies further suggest that titin-based forces are sufficient to account for the history-dependence of muscle force in lengthening^51^ and shortening^20^.

Previous studies have suggested that titin may bind to actin in active muscles^22,39,52,53^. Calcium-dependent binding of titin to actin can increase titin strain by preventing extension of compliant domains. However, binding alone cannot change titin force during the isometric force redevelopment period unless the binding site changes its location on the actin filament during isometric force redevelopment. If the binding site is maintained during isometric contraction, titin strain should not contribute to titin force during isometric force redevelopment following active shortening.

Assuming that titin functions as a linear spring, its force can be changed by adjusting its equilibrium length. Rassier and Herzog^18^ suggested that changes in the equilibrium length of a passive element during the isometric period following active shortening could explain the effect of active shortening on residual force enhancement. Unloading studies support the hypothesis that equilibrium length decreases during isometric force development^54^. The winding-filament hypothesis^51^ predicts changes in titin equilibrium length with increasing cross-bridge force. In the winding-filament hypothesis, strokes of cross bridges rotate actin filaments along their longitudinal axis based on actin’s double helix structure, and this rotation causes titin to be wound upon actin filaments. In particular, an increase in cross bridge force would decrease titin equilibrium length by longitudinally rotating the actin filament^51^. When cross-bridge force recovers during isometric force redevelopment, titin equilibrium length would be reduced by titin winding upon actin filaments. The reduction in titin equilibrium length should depend on the duration of isometric force redevelopment after active shortening.

The hypothetical change in titin equilibrium length during isometric force redevelopment following active shortening also suggests an alternative mechanism for residual force depression. In the winding-filament hypothesis^51^, the radial force of cross bridges rotates actin until the radial force of cross bridges is the same as the tensile force of titin. Active shortening should induce unwinding of titin at a rate that would depend on shortening velocity because muscle force depends on shortening velocity. Due to different rates of unwinding, the length of the titin free segment can differ because of the different shortening history, which in turn, can change the titin-based stiffness. The increasing length of the titin free segment would reduce titin force, which would also result in residual force depression.

For testing many hypotheses in muscle physiology, use of whole muscle preparations is often contra-indicated due to the increased complexity compared to single muscle fibers or fiber bundles because the extracellular matrix (ECM) of skeletal muscles, including collagen fibers, contributes substantially to skeletal muscle function^55,56^. The relatively much stiffer ECM contributes substantially to total muscle stiffness due to the parallel connection of ECM and muscle sarcomeres. However, a previous study found that the passive force of mouse soleus muscles after active shortening was independent of their shortening velocity^57^. This finding suggests that neither ECM nor intramuscular tendons contribute to residual force depression. Furthermore, the present study was carefully designed to produce minimal passive tension of the ECM by measuring only isometric muscle force and stiffness at L_0_. Therefore, the contribution of the ECM should be the same in all trials and independent of the previous shortening velocity.

Not only ECM and tendons contribute to overall muscle stiffness. Thick^35^ and thin^36^ filaments also contribute significantly to half-sarcomere stiffness^58^. However, we compared the ratios of force to stiffness measured at constant length and activation after different shortening histories. In order for thick or thin filament compliance to contribute to observed differences in the stiffness to force ratio at constant length and activation, the stiffness of these element must depend not only on the force but also on the shortening history. No previous studies have suggested that thick or thin filaments, tendons or ECM exhibit shortening-history-dependent force.

The relatively low correlation between isometric muscle force and stiffness for the ‘1/8 L_0_/s’ condition (Table 4, *r* = 0.84) might indicate a non-linear relationship. However, non-linear relationships including 2nd and 3rd order polynomials and exponential curves did not improve the correlation between muscle force and stiffness (Table 4). These results suggest that the relatively low correlation is due to variation among samples, rather than supporting a non-linear relationship. Additionally, the maximal force in the ‘no shortening’ condition is slightly above the linear fit (see Fig. 5). In the present study, muscle force and stiffness were measured at 10, 20, 80, and 1000 ms after active shortening. The interval between 80 and 1000 ms is fairly long, compared to the other times. If muscle force and stiffness had been measured at durations between 80 and 1000 ms, it would be possible to evaluate potential non-linearity more precisely. Future studies will further investigate the non-linearity of the relationship between *k*_*m*_ and *F*_*ISO*_.

**Table 4:** Coefficients and correlation for the linear and the non-linear relationships including 2nd order polynomial, 3rd order polynomial and exponential function between muscle force and stiffness during the isometric force redevelopment period following active shortening at three velocities (1/8, 1/2 and 2L_0_/s) and with no shortening.

Shortening velocity	1st polynomial		2nd polynomial		3rd polynomial		Exponential	
	$ax + b$		$ax^{2} + bx + c$		$ax^{3} + bx^{2} + cx + d$		$ae^{bx}$	
	coeff	*r*	coeff	*r*	coeff	*r*	coeff	*r*
No shortening	a = 1.01 b = − 0.067	0.98*	a = 0.78 b = − 0.019 c = 0.21	0.99*	a = 2.48 b = − 4.06 c = 2.89 d = − 0.31	0.99*	a = 0.16 b = 1.80	0.99*
1/8 L_0_/s	a = 0.65 b = 0.42	0.84*	a = 0.41 b = − 0.30 c = 0.78	0.84*	a = − 7.81 b = 18.73 c = − 14.21 d = 4.31	0.84*	a = 0.53 b = 0.71	0.84*
1/2 L_0_/s	a = 0.86 b = 0.21	0.98*	a = 0.69 b = − 0.099 c = 0.51	0.99*	a = − 3.66 b = 8.23 c = − 5.07 d = 1.56	0.99*	a = 0.37 b = 1.10	0.99*
2 L_0_/s	a = 0.96 b = 0.10	0.99*	a = 0.53 b = 0.30 c = 0.26	0.99*	a = 0.42 b = − 0.24 c = 0.74 d = 0.19	0.99*	a = 0.26 b = 1.45	0.99*

*p < 0.001.

We found different ratios of *k*_*m*_ to *F*_*ISO*_ during isometric force redevelopment following active shortening at different velocities. The greater *k*_*m*_ to *F*_*ISO*_ ratio caused by active shortening can potentially be explained by weakly-bound cross bridges that contribute to *k*_*m*_ but not to force, but cannot be explained by inhibition of strongly bound cross bridges. However, the weakly-bound cross-bridge hypothesis is not compatible with the reduced slope at the slower active shortening velocity or the absence of a non-linear relationship between *k*_*m*_ and *F*_*ISO*_ during isometric force redevelopment. To explain these observations, an alternative hypothesis is needed. One alternative hypothesis is that titin stiffness changes during active shortening and isometric force redevelopment. The winding filament hypothesis^51^ provides a potential mechanism for how titin stiffness is modulated by cross-bridge forces. However, since titin is unobservable due to its small (~ 4 nm) diameter^59–61^, direct evidence supporting the winding filament hypothesis has been elusive. In the absence of better techniques for observing sarcomere and protein structure during dynamic contractions, developing models that account for changes in muscle stiffness during shortening is essential to further the understanding of muscle mechanics in dynamic experiments with time-varying muscle length and velocity.

## Materials and methods

### Animals

Mice of the strain B6C3Fe a/a-Ttn^mdm^/J were obtained from the Jackson Laboratory (Bar Harbor, ME, USA) and a breeding colony was established in the animal care facility at Northern Arizona University (NAU). Soleus muscles from homozygous wild-type mice (n = 7; female = 2, male = 5; mouse mass = 23.3 ± 1.38 g; soleus muscle mass = 4.6 ± 0.48 mg) were used in this study. The Institutional Animal Care and Use Committee at NAU approved the experimental protocol and use of animals. The reporting of all animal experiments in the manuscript follows the recommendations in the ARRIVE guidelines and all experimental procedures were conducted in accordance with the American Veterinary Medical Association Guidelines for the Euthanasia of Animals.

### Muscle preparation

Wild-type mice were euthanized by isoflurane overdose, confirmed by cervical dislocation. Soleus muscles were isolated and the tendons at the muscle–tendon junction were tied with 4–0 silk suture as close as possible to the muscles to reduce contributions of extramuscular connective tissue. The muscles were attached to an inflexible hook at one end and to a servomotor length and force controller (Aurora Scientific, Inc., Series 300B, Aurora, ON Canada) at the other. Muscles were placed in an experimental chamber filled with mammalian Krebs–Ringer solution (in mM: 137 NaCl, 5 KCl, 1 NaH_2_PO_4_, 24 NaHCO_3_, 2 CaCl_2_, 1 MgSO_4_, and 11 dextrose, pH 7.4; buffered with 95% O_2_ and 5% CO_2_) at 22 °C.

Stimulation was achieved using two platinum electrodes placed parallel to the muscle in the chamber. For maximal tetanic stimulation, square wave pulses at 60 V were delivered to the muscles at a frequency of 75 Hz using a Grass S48 stimulator. To determine optimal muscle length (L_0_), muscles were activated using maximal tetanic stimulation and muscle length was adjusted until maximum isometric force was established. Maximum isometric force at L0, measured at the end of the experiments, was > 91.3 ± 2.2% of the maximum isometric force at L0 measured at the beginning of the experiments for all muscles.

### Experimental protocols

Maximum isometric force at 1.05 L_0_ was established for whole soleus muscles isolated from wild-type mice (Fig. 6, bottom). The muscles were actively shortened to L_0_ at three velocities (1/8 L_0_/s, ½ L_0_/s and 2 L_0_/s) and then held isometrically for four pause durations (10, 20, 80 and 1000 ms) which allowed isometric force redevelopment (Fig. 6, top). To measure instantaneous muscle stiffness, quick transients (0.9% L_0_ stretch at a speed of 3 L_0_/s) were conducted at the end of the pause^63^. The speed of the transient (3 L_0_/s) was based on a previous study^12^. For comparison, the muscle stiffness was also measured during isometric contractions at L_0_ without active shortening at similar durations after the onset of stimulation (10, 20, 80 and 1000 ms). The order of the different velocities and pause durations was randomized for each muscle. A period of 4.5 min rest was given between trials.

The present study was carefully designed to control for the contribution of the extracellular matrix (ECM) to force and stiffness. Muscle force and stiffness were measured only isometrically at L_0_, where the passive tension of the ECM is negligible^55,56^. Therefore, the contribution of the ECM to muscle force and stiffness should be small and similar in all of the trials, regardless of previous shortening velocity.

### Data analysis

Data were sampled at 4000 Hz and collected using a DAQ box (National Instruments, Austin, Texas, USA). Instantaneous muscle stiffness (*k*_*m*_) was calculated as the change in muscle force during the quick stretch, divided by the amplitude of the quick stretch (Fig. 6). *F*_*ISO*_ and *k*_*m*_ were measured at 10, 20, 80 and 1000 ms after the onset of isometric force redevelopment, and were normalized by the stiffness and force measured at 1000 ms without active shortening. The relative values were used for statistical analysis. Both relative force and relative stiffness were normally distributed (Shapiro–Wilk tests, all *p* > 0.22) and homoscedastic (Bartlett’s test, all *F* < 0.0077, all *p* > 0.99). Residual force depression was represented by the normalized value of *F*_*ISO*_ at 1000 ms after the onset of force development. Coefficients and correlations for the linear and the non-linear relationships including 2nd order polynomial, 3rd order polynomial and exponential functions between *k*_*m*_ and *F*_*ISO*_ were obtained for each condition including no shortening, 1/8L_0_/s, 1/2L_0_/s and 2L_0_/s using the *fit* function in MATLAB (Mathworks, R2020a, Natick, MA). The linear coefficients were reported for the slope and y-intercept of the relationship between *k*_*m*_ and *F*_*ISO*_. All data are shown as means ± S.D.

The relative force and stiffness data were analyzed in two different ways. First, two-way analysis of variance (ANOVA) was used to determine differences in normalized *F*_*ISO*_ and *k*_*m*_ among different shortening velocities and pause durations. Next, the effects of shortening velocity and *F*_*ISO*_ on *k*_*m*_ were tested using analysis of covariance (ANCOVA), with *F*_*ISO*_ as the main effect and active shortening velocity as the covariable. In both analyses, because all muscles experienced all combinations of shortening velocity and pause duration, estimating the interaction effect required the assumption that the between-muscle variance was negligible, which seemed reasonable because the data were normalized for each muscle by *F*_*ISO*_ at 1000 ms. Indeed, for both relative force and relative stiffness, the null probability that the means did not differ among muscles was > 0.78 (one-way ANOVA), and the null probability that the variance did not differ among muscles was > 0.99 (Bartlett’s test), supporting the assumption that the muscles can be treated as replicates. Lastly, the effect of shortening velocity on residual force depression was determined using one-way ANOVA. Tukey’s honestly significant difference (HSD) test was used to evaluate post hoc differences among means.

## Data Availability

The datasets used and analyzed in the current study are available from the corresponding author on reasonable request.
